# Supplementary material for: Nanopore metagenomic sequencing for detection and characterization of SARS-CoV-2 in clinical samples
Source: PLoS One. 2021 Nov 18;16(11):e0259712. doi: 10.1371/journal.pone.0259712 (PMC8601544; doi:10.1371/journal.pone.0259712)
Supplement: S1 Table — A. Primers and B. probes for RT-PCR testing performed at the BCCDC Public Health Laboratory and VGH. (DOCX) [file pone.0259712.s001.docx]

**S1 Table. A. Primers and B. probes for RT-PCR testing performed at the BCCDC Public Health Laboratory and VGH**

| **A. Primer Name** | **Sequence (5′ → 3′)** | **Target** | **Design** |
| --- | --- | --- | --- |
| **BCCDC RdRP** | | | |
| BCCDC RdRP Fwd | TGCCGATAAGTATGTCCGCA | RNA-dependent RNA polymerase, SARS-CoV-2 specific | Tracy Lee, BCCDC |
| BCCDC RdRP Rev | CAGCATCGTCAGAGAGTATCATCATT |  |  |
| **E-gene** | | | |
| E-gene_Fwd | ACAGGTACGTTAATAGTTAATAGCGT | Envelope protein, all SARS related viruses | Tracy Lee, BCCDC |
| E-gene_Rev | ATATTGCAGCAGTACGCACACA |  |  |
| **RNAseP** | | | |
| RNAseP F | AGATTTGGACCTGCGAGCG | Human ribonuclease P | Tracy Lee, BCCDC |
| RNAseP R | GAGCGGCTGTCTCAACAAGT |  |  |

| **B. Probe Names** | **Sequence (5′ → 3′)** |
| --- | --- |
| **RdRP gene** | |
| BCCDC RdRP probe | ***FAM***/TTGACACAGACTTTGTGAATG/*MGBNFQ* |
| **E-gene** | |
| E-gene _probe | ***Cy5***/ACACTAGCC/*TAO*/ATCCTTACTGCGCTTCG/IAbRQSp |
| **RNAseP** | |
| RNAseP probe | ***NED****/*TCTGACCTGAAGGCTC*/MGBNFQ* |

Mixed bases: **Y** = C or T, **K** = G or T, **R** = A or G
