# Supplementary material for: Nanopore metagenomic sequencing for detection and characterization of SARS-CoV-2 in clinical samples
Source: PLoS One. 2021 Nov 18;16(11):e0259712. doi: 10.1371/journal.pone.0259712 (PMC8601544; doi:10.1371/journal.pone.0259712)
Supplement: S2 Table — A. Primers, B. probes, and C. reaction conditions for Variant of Concern PCR testing. (DOCX) [file pone.0259712.s002.docx]

**S2 Table. A. Primers, B. probes, and C. reaction conditions for Variant of Concern PCR testing**

| **A. Primers** | | | |
| --- | --- | --- | --- |
| **Primer name** | **target** | **Sequence 5’-3’** | **Design** |
| N501Y_E484K-F1 | E484K | AGAGAGATATTTCAACTGAAATCTATCAGG | Tracy Lee, BCCDC |
| N501Y-R3 | E484K & N501Y | CCACAAACAGTTGCTGGTGC | Tracy Lee, BCCDC |
| N501Y-F2 | N501Y | AATTGTTACTTTCCTTTACAATCATATGG | Tracy Lee, BCCDC |
| WT_N501Y_block | N501Y | CCAAC+C+CA+C+T+A+A/3InvdT/ | Tracy Lee, BCCDC |
| E_Sarbeco_F1 | E-gene | ACAGGTACGTTAATAGTTAATAGCGT | Corman et al. |
| E_Sarbeco_R2 | E gene | ATATTGCAGCAGTACGCACACA | Corman et al. |

| **B. Probes** | | | |
| --- | --- | --- | --- |
| **Target** | **Probe Dye** | **Sequence 5’-3’** | **Design** |
| E484K_MGB-P1 VIC | VIC-MGB | CTTGTAATGGTGTTAAAGGT | Tracy Lee |
| N501Y-P_FAM MGB | FAM-MGB | CCAACCCACTTATGG | Tracy Lee |
| E_Sarbeco_probe | CY5-TAO | ACACTAGCCATCCTTACTGCGCTTCG | Corman et al. |

**C. Thermocycling program**

| **Step** | **Cycles** | **Temperature** | **Time** |
| --- | --- | --- | --- |
| RT | 1 | 50^0^C | 5 minutes |
| Enzyme Activation | 1 | 95^0^C | 20 seconds |
| Amplification | 40 | 95^0^C | 3 seconds |
|  |  | 60^0^C | 30 seconds |
