# Supplementary material for: Nanopore metagenomic sequencing for detection and characterization of SARS-CoV-2 in clinical samples
Source: PLoS One. 2021 Nov 18;16(11):e0259712. doi: 10.1371/journal.pone.0259712 (PMC8601544; doi:10.1371/journal.pone.0259712)
Supplement: S3 Table — Only pathogens with greater than 1% relative abundance in samples with at least 1000 bacterial reads were included. (DOCX) [file pone.0259712.s003.docx]

**S3 Table. Detection of potential pathogens in study samples.** Only pathogens with greater than 1% relative abundance in samples with at least 1000 bacterial reads were included.

| **Study ID** | **Bacterial Reads** | **Co-infection (% total bacterial/viral reads)** |
| --- | --- | --- |
| P1 | 9,553 | *Moraxella Catarrhalis* (7.4%) |
| P2 | 64,987 | *Haemophilus influenzae* (2.8%) |
| P4 | 24,479 | *Haemophilus parainfluenzae* (16.8%), *Neisseria meningitidis* (9.6%) |
| P7 | 3,896 | *Haemophilus influezae* (14.7%), *Haemophilus parainfluenzae* (6.9%) |
| P8 | 171,862 | Haemophilus parainfluenzae (1.6%) |
| P9 | 3,132,744 | *Haemophilus parainfluenzae* (1.1%) |
| P13 | 619,910 | *Haemophilus parainfluenzae* (4.5%), *Streptococcus pneumoniae* (2.7%) |
| P14 | 481,963 | *Moraxella catarrhalis* (31.6%) |
| P17 | 307,454 | *Haemophilus parainfluenzae* (3.5%) |
| P22 | 2,121 | *Neisseria meningitidis* (4.6%), *Klebsiella pneumoniae* (1.1%) |
| P27 | 2,862 | *Klebsiella pneumoniae* (69.3%) |
| P36 | 829 | *Staphylococcus aureus* (9.5%) |
| P38 | 7,376 | *Staphylococcus aureus* (10.1%) |
| N1 | 1,192 | *Staphylococcus aureus* (54.4%) |
| N5 | 55,465 | *Streptococcus pneumoniae* (59.5%) |
