# Supplementary figures and images for: Nanopore metagenomic sequencing for detection and characterization of SARS-CoV-2 in clinical samples
Source: PLoS One. 2021 Nov 18;16(11):e0259712. doi: 10.1371/journal.pone.0259712 (PMC8601544; doi:10.1371/journal.pone.0259712)

**
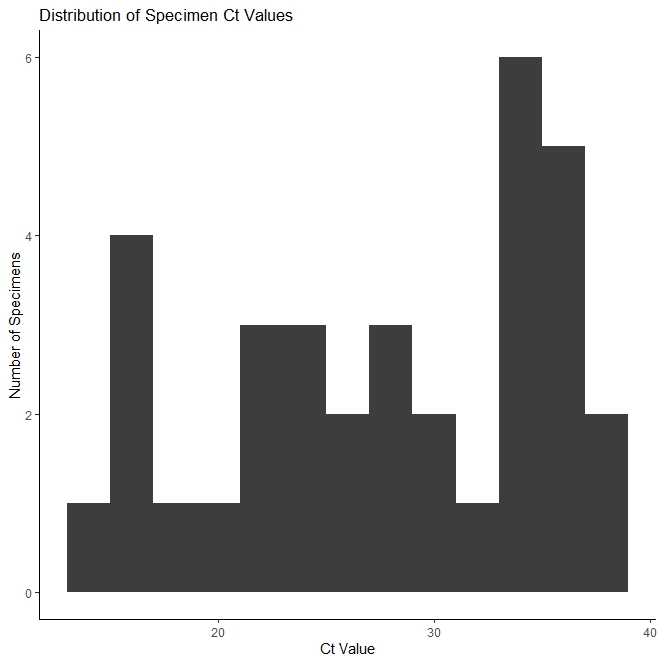
**

**S1 Fig.** Distribution of SARS-CoV-2 RT-qPCR positive study samples across Ct values

Supplement: S1 Fig — (DOCX) [file pone.0259712.s004.docx]

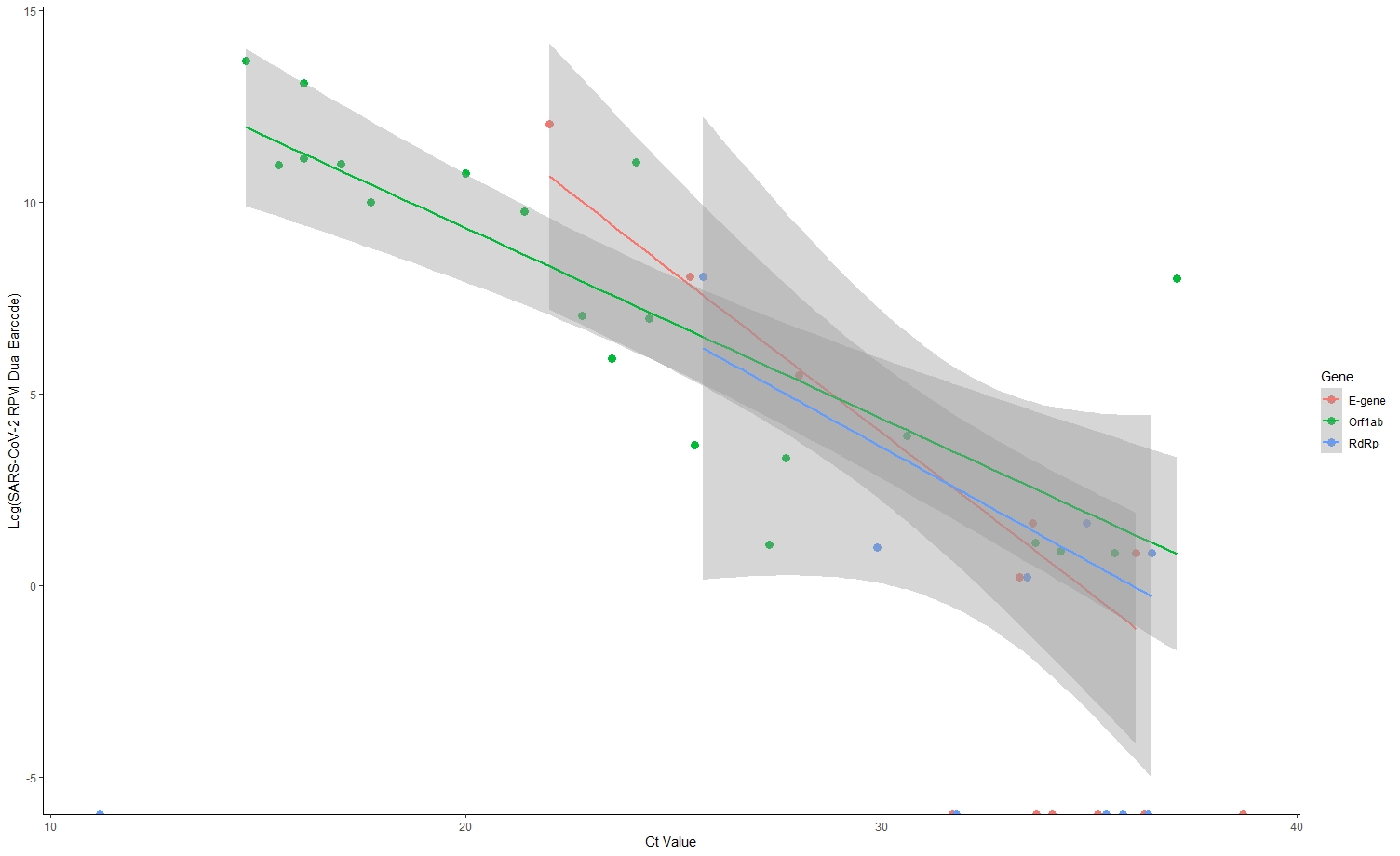


**S3 Fig.** Scatterplot of Log(SARS-CoV-2 RPM) against Ct value stratified by E-gene, ORF1ab, or RdRp.

Supplement: S3 Fig — (DOCX) [file pone.0259712.s006.docx]
