# Supplementary material for: Nanopore metagenomic sequencing for detection and characterization of SARS-CoV-2 in clinical samples
Source: PLoS One. 2021 Nov 18;16(11):e0259712. doi: 10.1371/journal.pone.0259712 (PMC8601544; doi:10.1371/journal.pone.0259712)
Supplement: S2 Fig — (DOCX) [file pone.0259712.s005.docx]

**
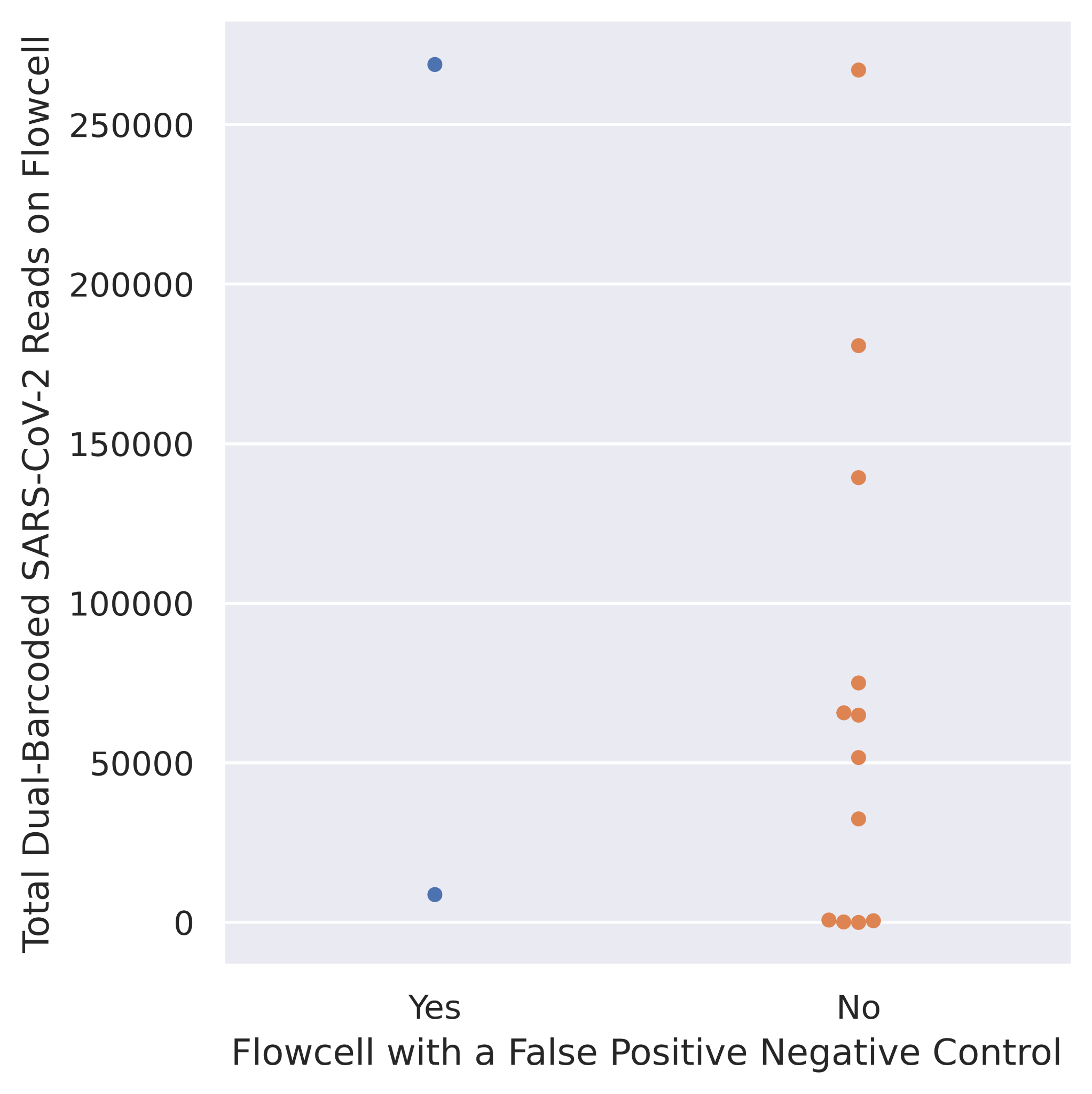
**

**S2 Fig.** Comparison of the total number of SARS-CoV-2 reads across all samples on a flowcell, stratified by flowcells with and without a false-positive negative control.
