# Supplementary material for: Nanopore metagenomic sequencing for detection and characterization of SARS-CoV-2 in clinical samples
Source: PLoS One. 2021 Nov 18;16(11):e0259712. doi: 10.1371/journal.pone.0259712 (PMC8601544; doi:10.1371/journal.pone.0259712)
Supplement: S4 Fig — Study samples are marked in red, publicly available genomes are black. (DOCX) [file pone.0259712.s007.docx]

**
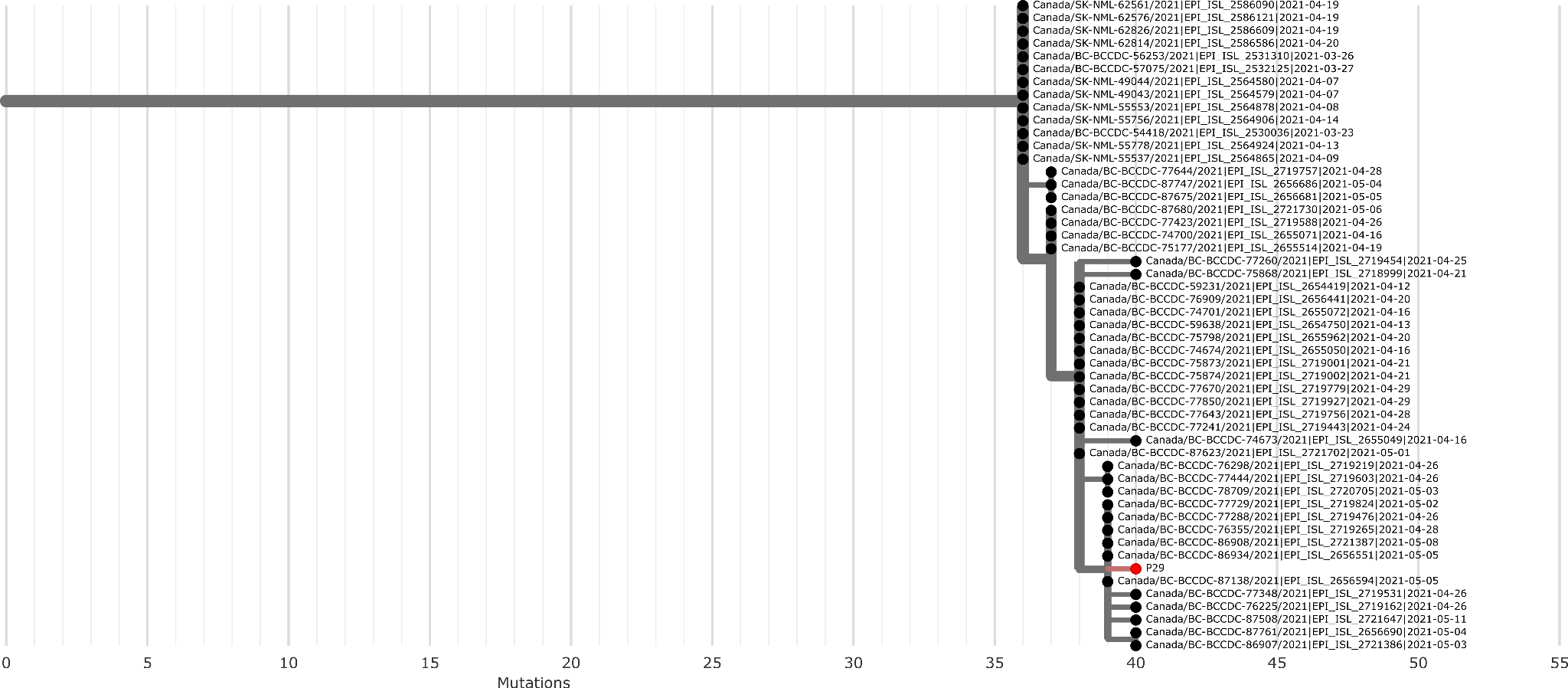

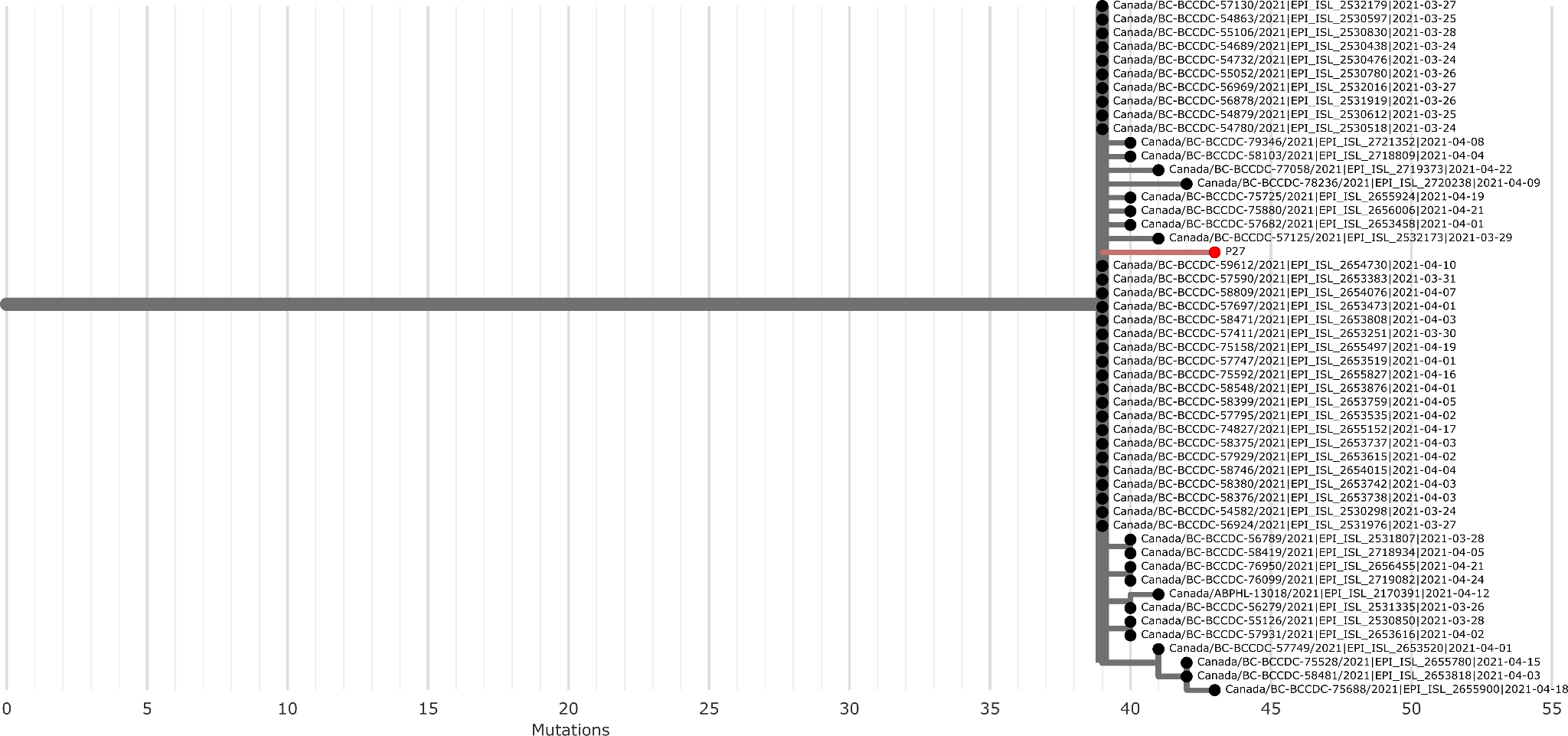

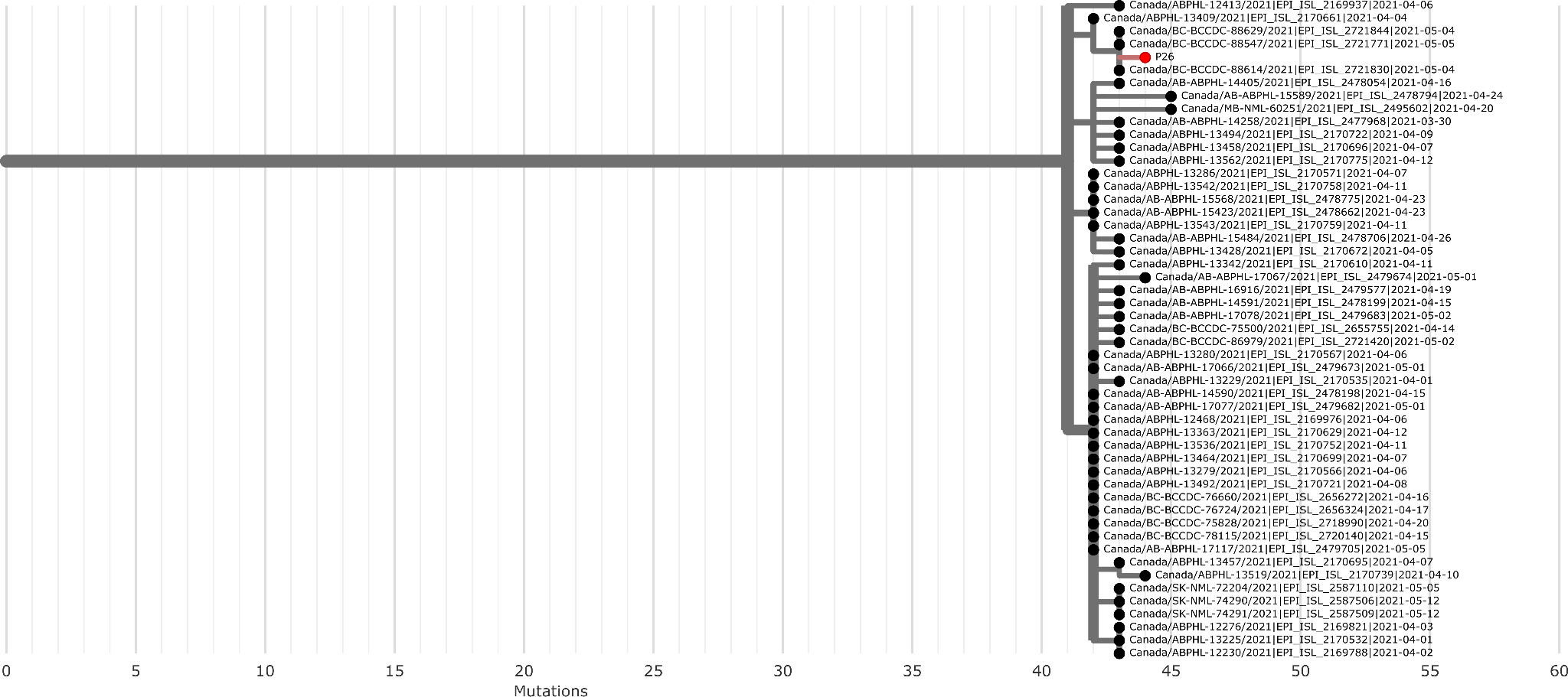

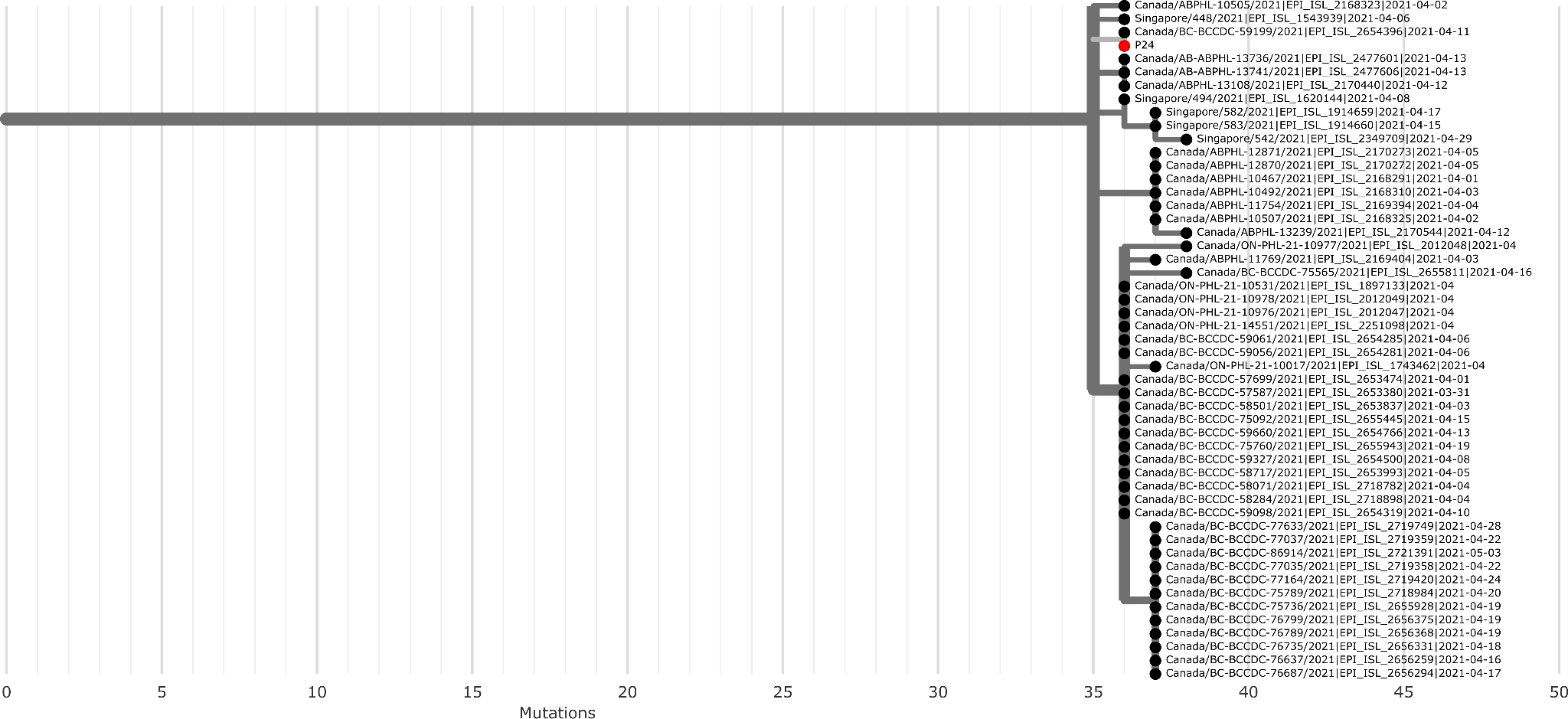

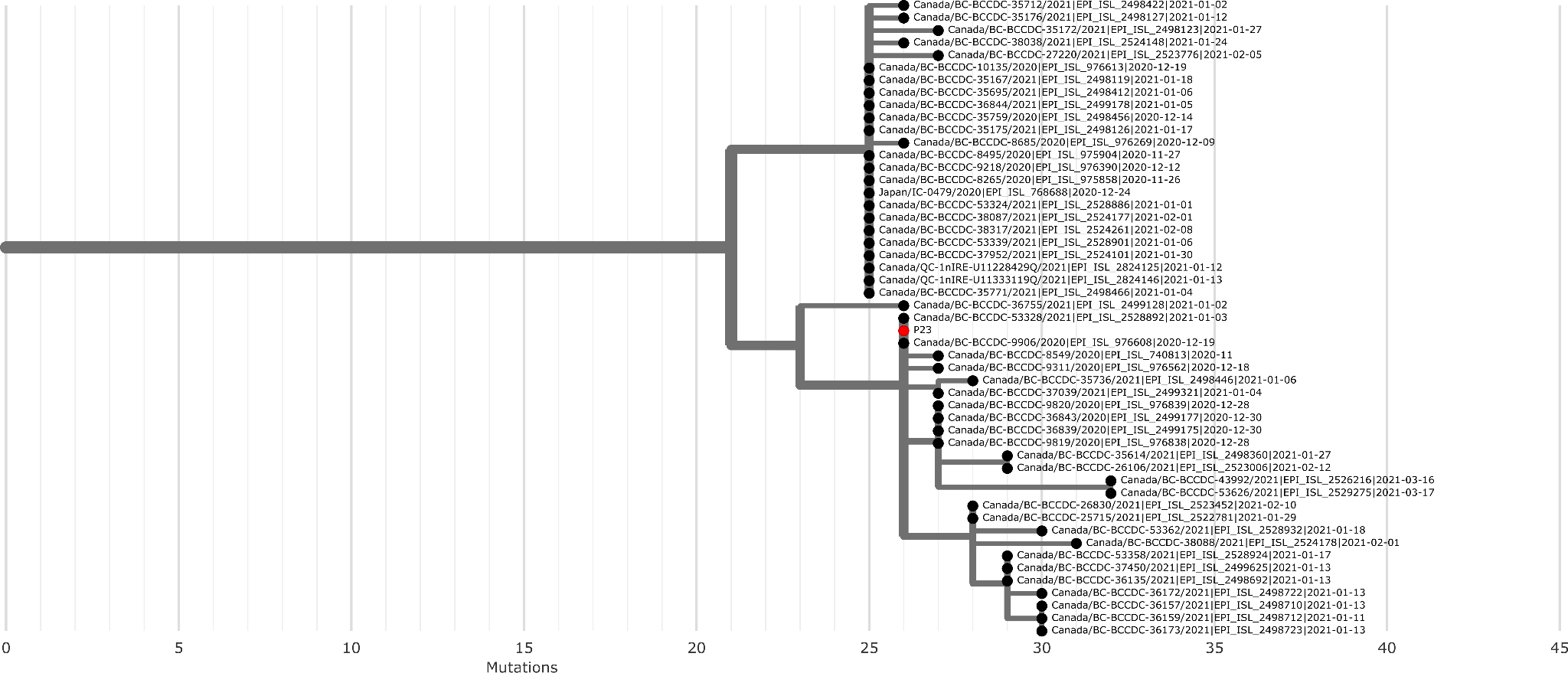

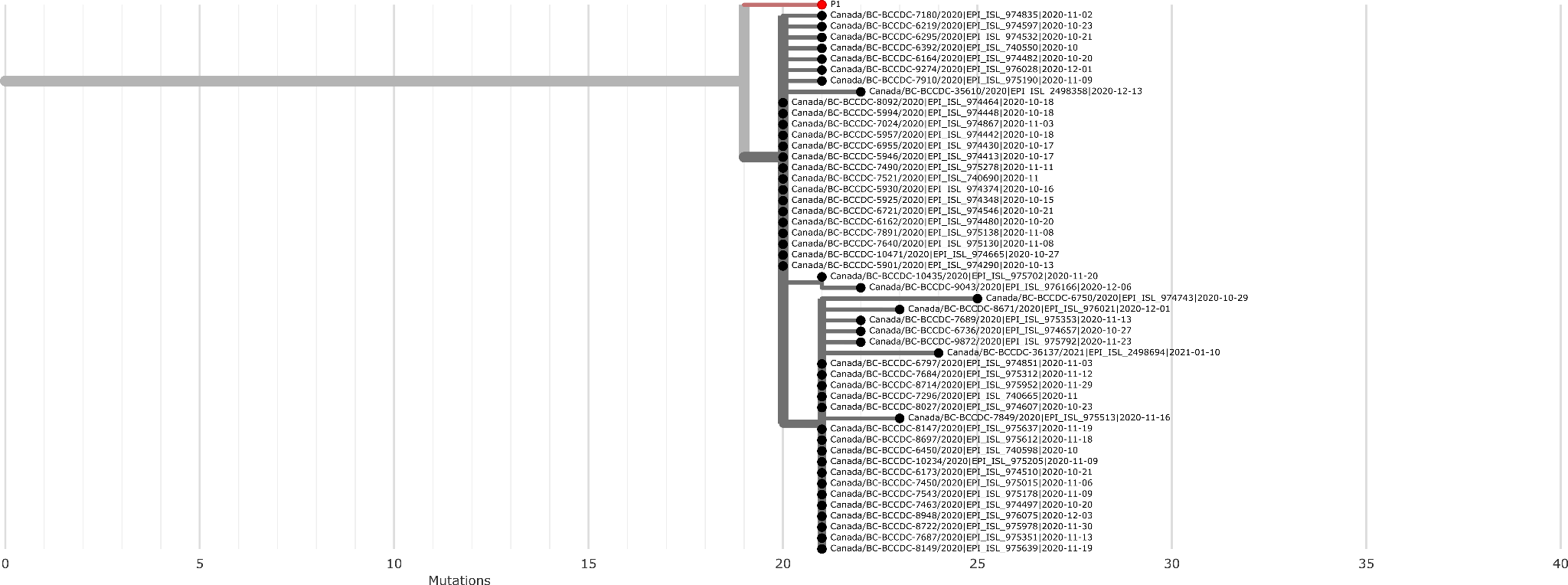

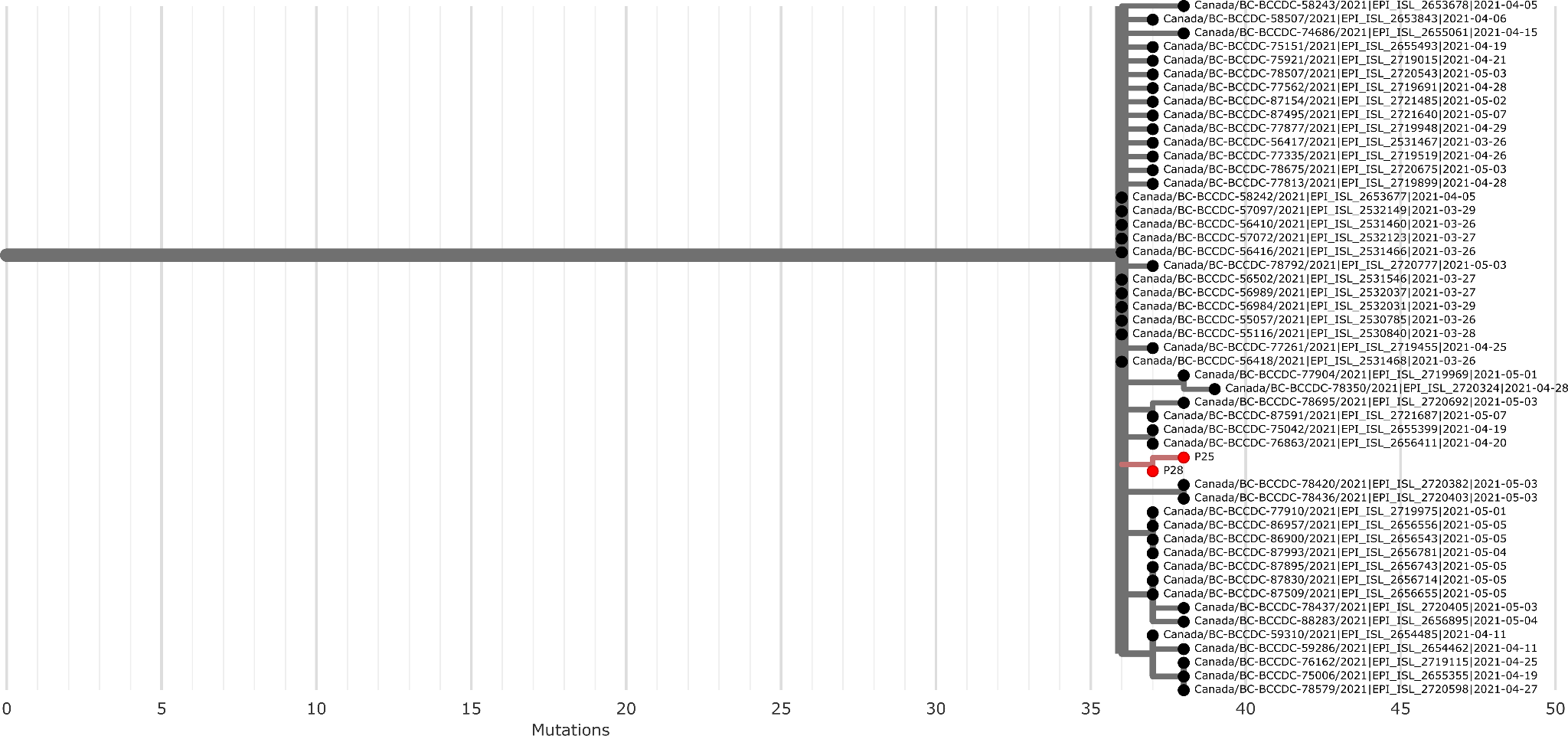

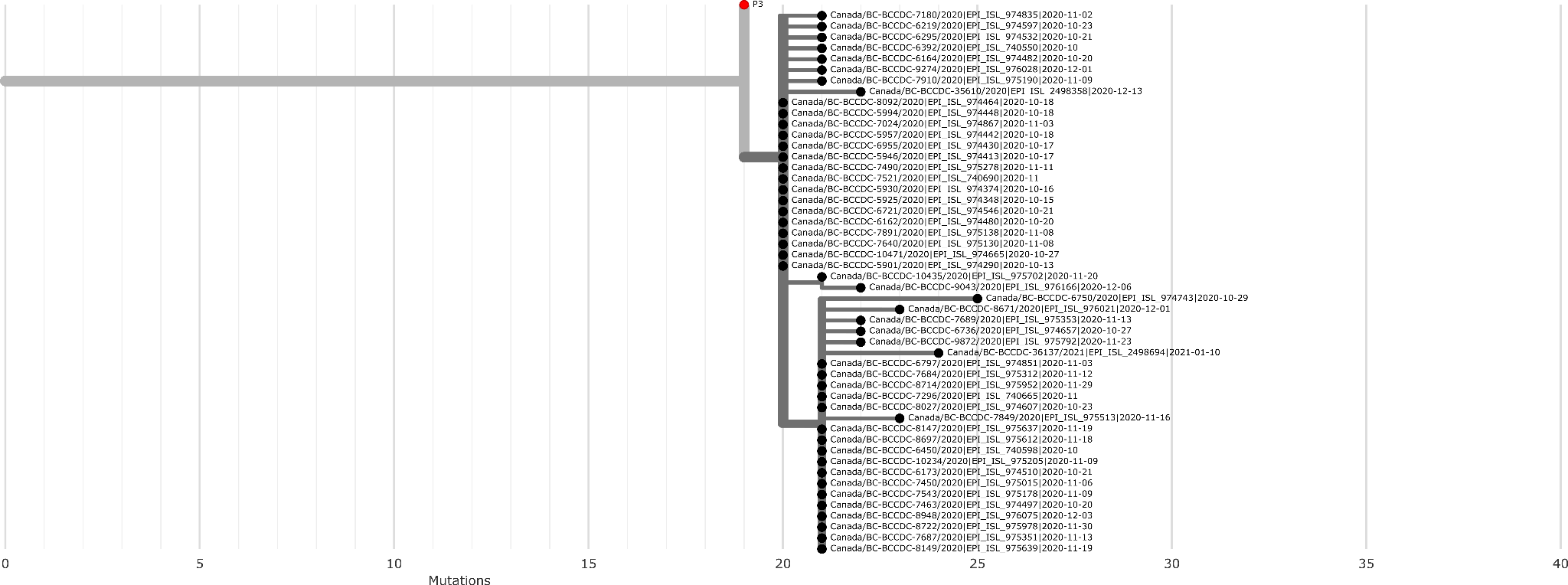

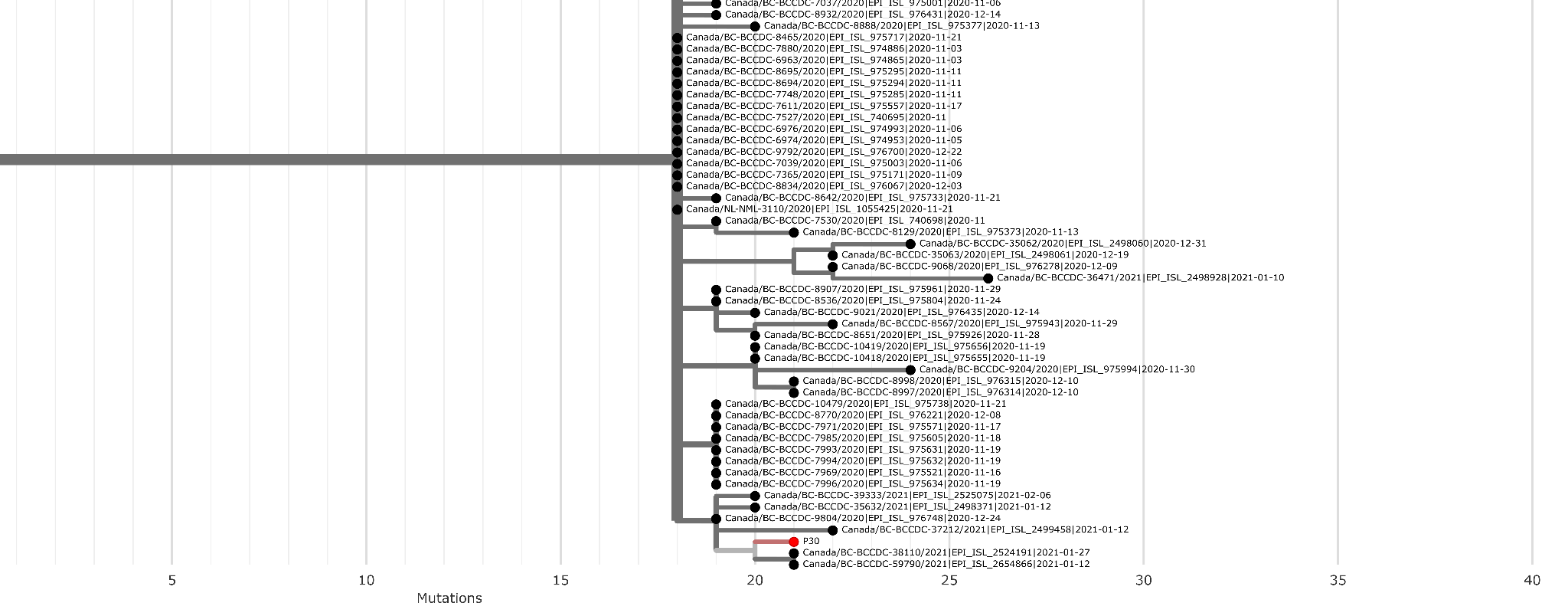
**

**S4 Fig.** Phylogenetic Subtrees of Study Samples Compared with 2,447,008 publicly available SARS-CoV-2 genomes. Study samples are marked in red, publicly available genomes are black.
